# Dose optimisation during imaging in radiotherapy

**DOI:** 10.2349/biij.3.2.e23

**Published:** 2007-04-01

**Authors:** P Ravindran

**Affiliations:** Christian Medical College, Vellore, India

**Keywords:** Animal irradiator, rodent irradiation, radiobiology, radiation beam measurement

## Abstract

The desire to increase the precision in radiotherapy delivery has led to the development of advanced imaging systems such as amorphous silicon (a-Si)-based electronic portal imaging, and kV and MV cone beam CT. These are used prior to the delivery of radiation to visualise the organ to be treated and to ensure that the patient setup and treatment delivery are accurate. However, little attention has been given to the dose received by adjacent normal tissues during these imaging procedures. Though these doses are very small compared to the dose delivered during radiotherapy, the involvement of normal tissues and the concern that these could increase the probability of stochastic effect, mainly the induction of secondary malignancy, cannot be ignored. This article reviews some work on the doses received during imaging in radiotherapy and the methods to optimise the same.

## INTRODUCTION

Recent technological developments have instilled considerable interest in advanced radiotherapy techniques such as Three Dimensional Conformal Radiotherapy (3D-CRT) and Intensity Modulated Radiotherapy (IMRT). These techniques have enabled dose escalation to the clinical target volume (CTV) and dose reduction to normal tissues as well as to the surrounding critical organs thus leading to better tumour control probability (TCP) and lower normal tissue complication probability (NTCP). The success of these techniques depends heavily on the accuracy in targeting the CTV, and achieving the aimed sparing of the normal tissues. Conventionally, external markers made on the patient surface are used to direct the radiation beam to the target volume. This could result in geographical miss as the relationship between the external marker and the CTV could have been lost during the time between planning and the treatment, and during the course of the treatment. In order to achieve accurate targeting during treatment, the organ to be treated is visualised using portal imaging. Portal imaging is the use of a therapeutic X-ray or gamma ray beam to form an image of the area being irradiated and its main application is to analyse the patient setup during treatment and account for the uncertainty in treatment delivery. It is well known that the images produced with Megavoltage (MV) beam suffer from low subject contrast compared to the images produced with kV X-ray beams. This is mainly due to the fact that the MV beams interact with the patient by Compton scattering that depends on the density rather than the atomic number. In addition to this, many other factors contribute to the poor quality of images in portal imaging and these include the performance of image receptor, scatter due to patient thickness, source size, etc. [1].

Portal images have been defined as three types: localisation radiograph, verification film and double exposure [2]. X-ray film is the traditional medium for portal imaging. Historically, portal imaging has been performed with industrial films [3] mainly for complex-shaped beams such as the one used in Mantle Technique for Hodgkin’s disease. A few centres have also been using the Computed Radiography (CR) technology with the Photostimulable Phosphor Plate (PSP) for MV portal imaging in radiation therapy [4]. The main advantage of this method is that the images are made available in digital format. Electronic portal imaging (EPI) was introduced in the early 1980s when the late Norman Baily demonstrated the use of a fluoroscopic system to acquire MV transmission images. Since then electronic portal imaging devices have undergone significant developments from CCD-based imaging devices to a-Si flat panel devices. Electronic portal imaging devices (EPID) have many potential advantages over traditional X-ray films for portal imaging. The images obtained are immediately available and can be used interactively to adjust patient or field position during radiotherapy. The digital images aid image processing, contrast enhancement and image matching. Moreover, digital archiving saves space and allows for rapid recall of images over a network [3]. These developments in portal imaging have resulted in the development of 3D imaging for positioning.

The advantage of obtaining full volumetric information with single rotation of the source and the flat panel detector with cone beam CT has led to its introduction as an image guidance system in radiation therapy. Cone beam CT generated with the MV beam is used to obtain patient setup information in 3D by registering these images with the planning CT images in radiation therapy. A few vendors provide cone beam CT obtained with kV beam for setup verification. This has been made possible by having a kV X-ray tube and a flat panel detector 90° to the treatment beam [5].

The requirement of radiation protection as per the international basic safety standards (BSS 115) is that “any medical exposure should be justified by weighing the diagnostic or therapeutic benefits they produce against the radiation detriment they might cause by taking into account the benefits and risks of available alternative techniques that do not involve medical exposure”. In radiation therapy, it is generally assumed that the potential of radiation dose delivered during imaging for verification and localisation does not add to the patient’s burden because the doses from such exposures are very small compared with the intended therapy dose. This is true when considering imaging of the target volume that receives the intended prescribed dose. However, imaging during radiotherapy for setup verification results in dose exposure to normal tissues outside the tumour volume. In this paper, the dose received due to portal imaging, image guidance techniques, such as MV and kV cone beam CT, and the methods that have been suggested to optimise the dose to normal tissues during imaging in radiotherapy, are reviewed.

## DOSE OPTIMISATION DURING PORTAL IMAGING IN RADIATION THERAPY

It has been concluded from early studies that portal films are essential for accurate delivery of radiation therapy, and frequent filming may be required to decrease the frequency of localisation and field design error [2]. Portal images are acquired either by single exposure technique or by double exposure technique. In single exposure technique, the treatment beam is used to image the region to be treated when adequate landmarks are available for verification within this region. The dose delivered during this single exposure portal imaging is usually adjusted from the treatment dose and this does not deliver dose to normal tissues. When adequate landmarks are not available within the treatment region, a double exposure technique is used. In this double exposure technique, a field at least 5cm larger than the area to be treated is also imaged in addition to the region to be treated. This results in delivering dose outside the tumour volume and thus increases the probability of stochastic effect. The risk of cancer induction is additive and the concomitant dose from the double exposure portal images adds to the dose received by the patient due to leakage and scatter radiation [6]. Several studies have been performed to increase the image quality during portal imaging and thus reduce the dose during portal imaging. Crooks and Fallore have estimated the dose during the portal imaging with three different films viz. CEA TLF, CEA TVS and Kodak EC-L films and the dose during portal imaging is estimated as 1.2 cGy, 15.9 cGy and 1.5 cGy for the CEA TLF, CEA TVS and Kodak EC-L films, respectively [7]. The authors have concluded that the CEA TVS film could be used for single exposure, where it could be taken out during the exposure (treatment) and CEA TLF or the Kodak EC-L films for double exposure as they require very low dose to get optimally-exposed images.

**Figure 1:**
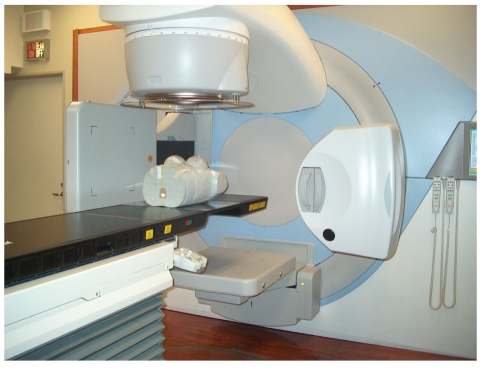
(a) Single exposure port film obtained with Computed Radiography (CR) for 6 MV X-rays; (b) Double exposure port film obtained with Computed Radiography (CR) for 6 MV X-rays.

A few authors have tried portal imaging with computed radiography (CR), a non-film-based system, used to obtain high quality portal images. In this system, the film is replaced with a photostimulable phosphor plate [8]. In addition to the advantages of digital imaging, this technique produces excellent images with radiation exposures of 1 and 2 monitor units (MU) only. The use of CR for portal imaging is also being tried in the author’s institute where PSP plates are used with a CR reader for obtaining portal images. Single and double exposure images obtained with 1 MU/exposure are shown in Figure 1a and Figure 1b, respectively.

Jaffray *et al* have suggested a method to reduce the dose during double exposure and enhance the image quality by using a dual-beam system consisting of a kV X-ray tube mounted on the gantry of a medical linear accelerator thus mixing low- and high-energy beams for double exposure technique [9]. Both the kV and MV images are collected with a fluoroscopic imaging system that uses a low-noise CCD camera to accumulate the light emitted from a phosphor screen. The authors have concluded that the quality of the dual-beam image is similar to the prescription (simulation) image, contains a larger anatomical region, and delivers a lower integral dose to the patient.

Radiation dose delivered during double exposure portal imaging for pediatric radiotherapy has been evaluated by Kudchadker *et al* by conducting a retrospective review of the port film dose for 56 consecutive pediatric patients who underwent definitive radiation therapy [4]. The mean total port dose varied from a maximum of 46 cGy for the brain to a minimum of 17 cGy for the thorax. The mean total port dose as a percentage of prescribed dose was less than 1.25% for all locations in this study. However, most of the port dose is a result of the open-field dose from the double-exposure technique. Kudchadker *et al* suggest that care should be exercised while exposing port films of pediatric patients to minimise both the number of films and the radiation dose without compromising the quality of treatment delivery. The specific suggestion of the authors is to minimise the number of monitor units used to image regions outside the treatment field to reduce the risk of development of secondary neoplasms.

### Development of Electronic Portal Imaging devices and Cone Beam CT systems

The requirement for better visualisation of MV images and enhanced image quality for verification of setup for conformal radiotherapy has led to the development of online electronic portal imaging devices, which have replaced the conventional film-based portal imaging [10]. The main advantage of this type of portal imaging over film is the immediate (online) availability of images that enables verification and correction of patient setup before treatment.

**Figure 2:**
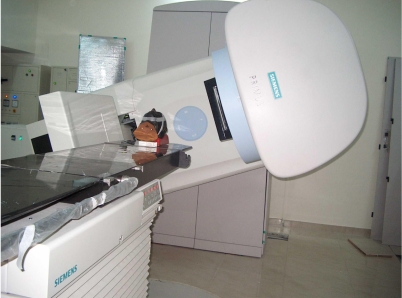
Primus linear accelerator with a-Si flat panel electronic portal imaging device.

**Figure 3:**
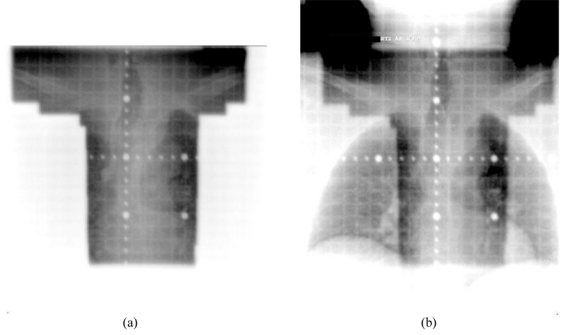
kV X-ray source and flat panel detector fixed at 90° to treatment beam of a linear accelerator for image guidance with kV cone beam CT.

The availability of digital images from the EPID makes image processing, contrast enhancement and automatic comparison with planning images (simulator images or DRR) possible. The development of the EPID has undergone several significant changes starting from video camera- and mirror-based EPIDs, to the recent a-Si based flat panel EPIDs. The construction, development and the physics of these portal imaging devices have been discussed in several publications [1, 3, 11]. The other major advantage with the a-Si flat panel EPIDs is the considerable low MUs required to produce the portal images. A primus linear accelerator fitted with an a-Si flat panel portal imaging system is shown in Figure 2. A portal image acquisition mode has also been developed for the PortalVision™ that allows one to take portal images with reduced dose while keeping good image quality [12]. Moreover, the introduction of the a-Si flat panel imaging devices and the interest to have 3D verification images have led to the development of cone beam CT-based Image Guided Radiotherapy system (IGRT). The availability of full volumetric information of the patient anatomy with single rotation of the source and the detector has led to the development and clinical implementation of both the MV and kV cone beam CT for image guided precision radiotherapy. MV cone beam CT has been developed using the treatment beam and the EPID [13-15]. In order to increase the image quality kV cone beam CT has been developed with a kV X-ray tube and an a-Si flat panel detector fixed at 90^o^ to the MV source [5, 16]. An Elekta linear accelerator with integrated kV CBCT system is shown in Figure 3. The imaging performance of both kV and MV cone beam CT has been compared [17].

### Dose during MV and kV CBCT

Although the clinical use of cone beam CT in image guidance has improved the accuracy in patient setup and radiation delivery, the dose delivered during the image guidance is of significant concern, as the volume of the tissue irradiated is much more than the volume to be treated and several times higher than the volume irradiated during the double exposure technique of portal imaging. Moreover, repeated imaging using the cone beam CT through several fractions during the course of the treatment is likely to result in a significant dose to normal tissues that can increase the probability of stochastic effect.

#### MV cone beam CT

MV cone beam CT is simple to use, as it does not require additional hardware except for a-Si flat panel detector with high detection quantum efficiency. However, relatively lesser image quality compared to kV cone beam CT images and radiation dose during the cone beam CT are of great concern. Initial attempts to generate MV cone beam CT with about 90^o^ projections resulted in a central dose of about 90 cGy [13]. As one cannot employ full-field imaging on a regular basis with the MV cone beam CT doses mentioned above, attempt has been made to image the region of interest (GTV/PTV) using conformal beams and to improve the image quality with a-Si flat-panel EPID that has higher detective quantum efficiency [15]. The dose during the MV cone beam CT was reduced by Seppi *et al* by developing an image receptor that uses a flat-panel imaging system consisting of a conventional flat-panel sensor attached to a thick CsI scintillator [18]. The scintillator consists of individual CsI crystals 8 mm thick and 0.38 mm by 0.38 mm pitch. With this, projection images could be obtained with one accelerator pulse delivering as little as 0.023 cGy per image. They could generate MV CT images with soft tissue contrast with irradiations as small as 16 cGy. Further dose reduction during MV cone beam CT has been achieved by windowing the dose-pulse rate of 6 MV Primus accelerator beam to expose an a-Si flat panel by using only 0.02 to 0.08 MUs per image and produce low-noise 3D MV cone beam CT images without pulsing artifacts with a total delivered dose that ranged from 5 to 15 cGy [19].

#### kV cone beam CT

Imaging using a kV beam is superior to the MV imaging as photoelectric interaction, which depends on the atomic number, is dominant in this energy range and hence gives better subject contrast resulting in clear differentiation between bone and tissue. Although the dose to the patient due to a kV image-guided radiotherapy is small compared to that of the MV image guidance, a large volume of the normal tissue is involved in the cone beam CT imaging and the repeated use of this modality for image guidance on a daily basis may contribute significant dose to normal tissue. The dose during a kV cone beam CT and the dose at the centre and surface of the body phantom have been estimated as 1.6 cGy and 2.3 cGy for a typical imaging protocol using full rotation scan, with a technique setting of 120 kVp and 660 mAs [20]. Similarly, the dose to the surface of the eyes during a kV cone beam CT head scan and the dose to the surface of the breast and the contra-lateral breast during a kV cone beam CT breast scan have been estimated with humanoid phantoms, and methods have also been suggested to reduce the dose during a kV cone beam CT [21]. Using a lesser number of projections to reduce the dose and the impact on the image quality has also been studied [20]. The possibility of achieving an ultra-low dose of about 1 mGy per scan has been reported by attempting to generate low exposure volumetric X ray CT by reducing the number of exposures (projections) [22].

## CONCLUSION

Imaging during radiation therapy has a significant role as it reduces the setup error and avoids geographical miss. Portal imaging and 3D image guided radiotherapy with cone beam CT have increased the accuracy of treatment delivery considerably. However, one should remember that a significant amount of normal tissue is irradiated in this process and could increase the probability of stochastic effect. Hence, it is necessary to develop protocols to optimally use the imaging techniques for treatment delivery in radiotherapy that could significantly reduce the risk of stochastic effect.
